# Investigating Texture and Freeze–Thaw Stability of Cold-Set Gel Prepared by Soy Protein Isolate and Carrageenan Compounding

**DOI:** 10.3390/gels10030204

**Published:** 2024-03-18

**Authors:** Zhuying Wang, Zhenhai Yu, Shuanghe Ren, Jun Liu, Jing Xu, Zengwang Guo, Zhongjiang Wang

**Affiliations:** 1 College of Food Science, Northeast Agricultural University, Harbin 150030, China; 15046617734@163.com (Z.W.); ren10180132@163.com (S.R.); 2 Heilongjiang Province Green Food Science Institute, Harbin 150028, China; hyzh1188@sina.com; 3 Kedong Yuwang Co., Ltd., Qiqihaer 161000, China; liujun@yuwangcn.com; 4 College of Arts and Sciences, Northeast Agricultural University, Harbin 150030, China; xujing@neau.edu.cn; 5 National Grain Industry (High-Value Processing of Edible Oil Protein) Technology Innovation Center, Harbin 150030, China

**Keywords:** soy protein isolate, carrageenan, cold-set gel, freeze–thaw stability

## Abstract

In this study, the purpose was to investigate the effects with different concentrations of carrageenan (CG, 0–0.30%) on the gel properties and freeze–thaw stability of soy protein isolate (SPI, 8%) cold-set gels. LF-NMR, MRI, and rheology revealed that CG promoted the formation of SPI-CG cold-set gel dense three-dimensional network structures and increased gel network cross-linking sites. As visually demonstrated by microstructure observations, CG contributed to the formation of stable SPI-CG cold-set gels with uniform and compact network structures. The dense gel network formation was caused when the proportion of disulfide bonds in the intermolecular interaction of SPI-CG cold-set gels increased, and the particle size and zeta potential of SPI-CG aggregates increased. SG20 (0.20% CG) had the densest gel network in all samples. It effectively hindered the migration and flow of water, which decreased the damage of freezing to the gel network. Therefore, SG20 exhibited excellent gel strength, water holding capacity, freeze–thaw stability, and steaming stability. This was beneficial for the gel having a good quality after freeze–thaw, which provided a valuable reference for the development of freeze–thaw-resistant SPI cold-set gel products.

## 1. Introduction

Soy protein isolate (SPI) is extensively used in food production. This is due to the fact that SPI offers diverse biological activities, such as reducing blood pressure and mitigating osteoporosis [1]. As a result, to meet the various needs of consumers, SPI is widely employed as an ingredient in the development of functional high-protein foods. In addition, SPI has diverse functional properties, such as good emulsifying, foaming, and gel properties. A major characteristic of SPI is the gel, which imparts unique texture characteristics to food, such as elasticity and hardness. Therefore, gel food has great sensory properties and commercial value [2,3]. Usually, SPI gel formation methods are mainly divided into heat-induced gel and cold-set gel. SPI cold-set gel not only plays a role in traditional food processing (Chiba tofu) [4], but also shows good application characteristics in drug delivery (beta-carotene) [5]. Therefore, SPI cold-set gel has attracted more and more attention recently.

Protein cold-set gel is primarily prepared in two stages. In the first step, soluble protein aggregates are produced by preheating a native protein solution at a pH outside the isoelectric point and at a low ionic strength. In the second step, when the aggregates are cooled, a coagulant is added to the aggregates to reduce intermolecular repulsion and promote gelation [6]. The current gelation coagulants of soy protein include inorganic salts (KCl, CaCl_2_, NaCl), transglutaminase enzyme, or glucose-δ-lactone (GDL). There is a wide range of applications for GDL as an acid coagulant—for example, lactone tofu. However, it is difficult to avoid the product quality declining, caused by freezing and cold storage during the processing and transportation of protein cold-set gel food. During the freezing process, the water present in product items undergoes a phase transition and turns into ice. When thawing, moisture tends to be released from the network of protein gels [7]. These changes will lead to the deterioration of product texture and an overall decline in quality, which results in consumer unacceptability. Thus, it is a challenging but necessary endeavor to develop protein cold-set gels that minimize the negative effects caused by freeze–thaw. A variety of methods are available to improve the freeze–thaw stability of protein, including ultrasonic treatment, radiation-assisted Maillard reaction, pH modification, and adding polysaccharides [8,9,10,11]. Relatively, adding polysaccharides can have a better effect on the freeze–thaw stability and will not affect the nutritional characteristics and biological activity of protein.

As an anionic polysaccharide, carrageenan (CG) is a linear and sulfated polysaccharide formed from D-galactose and 3,6-anhydro-D-galactose. A number of studies have shown that CG can act as a low-calorie dietary fiber, which is often used to increase the freeze–thaw behavior of meat gels. For example, during repeated freeze–thaw cycles, CG can be used as a protective agent for the protein, lipid, and moisture migration of *Litopenaeus vannamei* [12]. It effectively enhances its freeze–thaw resistance and improves its quality after freeze–thaw cycles. Therefore, CG provides an important method to enhance the gelling characteristics and texture properties of protein gels. Altogether, it broadens the range of applications for protein gels in the food industry. Furthermore, studies suggest that Guar gum and xanthan gum can improve the freeze–thaw stability of SPI emulsion gels [10]. Therefore, based on a large literature report, we believe that CG is expected to improve the freeze–thaw stability of SPI cold-set gel. It may play a guiding role in the development of SPI-CG cold-set gel-related foods, such as the cold chain transportation of lactone tofu and the embedding of heat-sensitive nutrients [13,14].

In this work, the purpose was to evaluate the influence of different CG concentrations on SPI cold-set gels, to improve SPI-CG cold-set gelation properties and freeze–thaw stability. Thus, we investigated the structure of SPI cold-set gels after adding CG, through LF-NMR, MRI, rheology, and microstructure. Through the changes in the intermolecular interaction of SPI-CG cold-set gel and the particle size and zeta potential of SPI-CG aggregates, we explored the influence of CG on SPI-CG gel networks. We also evaluated the gel properties of SPI cold-set gel after adding CG through gel strength, water holding capacity, freeze–thaw stability, and steaming stability. In addition, this study could provide useful information for understanding the mechanisms of improving SPI acid-induced cold-set gelation by CG. It could also extend the range of applications of SPI–CG cold-set gels.

## 2. Results and Discussion

### 2.1. Water Distribution by LF-NMR and MRI

**Figure 1:**
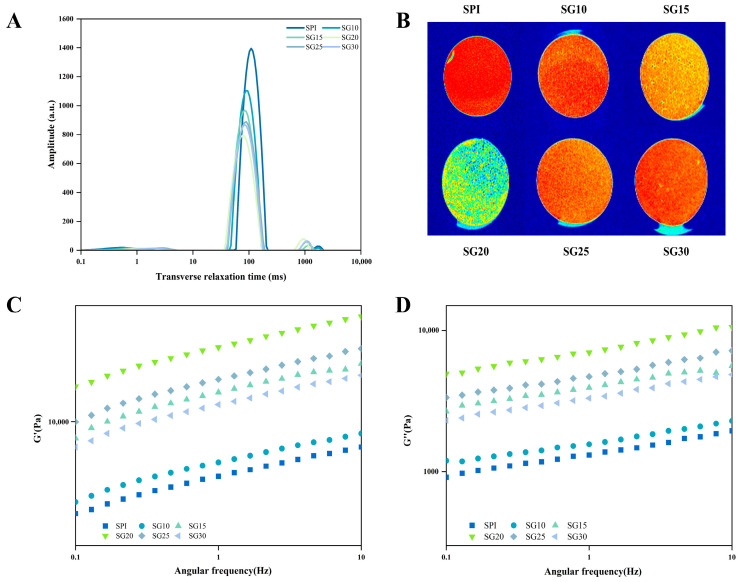
Plotting of (**A**) the curve of T_2_ relaxation time; (**B**) magnetic resonance images; (**C**) storage modulus G′; (**D**) loss modulus G″ in the SPI-CG composite gels.

LF-NMR can be used to determine the water-holding capacity of SPI-CG cold-set gels, the distribution and transport patterns of water, and the quality properties of network structures in SPI-CG gels. In Figure 1A, it can be observed that the SPI-CG cold-set gels exhibited a weak peak at around 0–10 ms (T_2b_ and T_2b−1_), an intense peak at around 50–150 ms (T_21_), and a weak peak at around 500–2000 ms (T_22_), indicating the presence of three water relaxation curves. This represented the three water states in the gel system, namely, bound water, immobile water, and free water. Bound water refers to water that is tightly bound to the carbonyl, amino, and carboxyl groups of proteins by hydrogen bonds. Gels immobilized water mainly refers to water retained within their spatial network structures. There is no restriction on free water moving within the SPI-CG network [15]. The relaxation time peak areas (T_2_), segmented relaxation times (T_2b_, T_2b−1_, T_21_, and T_22_), and the proportions of segmented relaxation times (P_2b_, P_2b−1_, P_21_, and P_22_) that belonged to SPI-CG cold-set gels are summarized in Table 1. Figure 1A shows that the main water component of SPI-CG cold-set gels was T_21_. When the CG concentration increased, the peak of T_21_ shifted to lower relaxation times. In the presence of a 0.20% CG concentration, the peak of T_21_ was shifted to a higher relaxation time, resulting in the lowest relaxation times. It appears that the mobility of SPI-CG cold-set gels’ immobile water may be reduced by adding the correct concentration of CG. It indicates that the addition of CG can improve the binding ability to immobilize water [16]. This phenomenon may be closely related to the density of SPI-CG cold-set gels’ network structure. An appropriate CG concentration due to the SPI-CG cross-linking led to a dense gel network formation, resulting in the immobilizing water ability of SPI-CG cold-set gels being improved. Zhao et al. showed that the addition of Tremella fuciformis polysaccharide promoted the formation of a dense gel network, resulting in the reduced fluidity of water molecules and the tighter water binding of SPI-CG gels. This was consistent with our analysis [17]. When the addition of CG was excessive, the space steric effect caused voids increasing in size in gel networks, resulting in the immobilizing water ability of SPI-CG cold-set gels being decreased. Table 1 shows how the proportion of P_22_ in gels initially increased before decreasing and the proportion of P_21_ in gels initially decreased before increasing. It indicates that the addition of CG promoted the mutual conversion between immobilized water and free water in SPI-CG cold-set gels. The conversion of immobilized water to free water may be related to dehydration in the SPI-CG gel network [18].

With the help of magnetic resonance imaging (MRI), the hydrogen proton content (water content) of the SPI-CG cold-set gels could be further analyzed, resulting in more red pseudo-color images for hydrogen protons in the given area [19]. Figure 1B illustrates the pseudo-color maps of the SPI-CG cold-set gels. It shows that SPI had more red areas. The SPI-CG cold-set gels were gradually yellowing followed the addition of CG. The SPI-CG cold-set gels turned to green when the concentration of CG was increased to 0.20%. Continuously increasing the amount of CG added, the color of the SPI-CG cold-set gels turned to red again. It indicates that the addition of CG caused water precipitation, and SG20 had the lowest water content. Hence, the MRI results were similar to the T_2_ results. This may be due to the addition of CG promoting a denser gel network, thereby reducing the formation of interconnecting water channels in SPI-CG cold-set gels. Moreover, the precipitation of water due to the dense gel network was conducive to increasing the effective concentration of SPI-CG aggregates, resulting in higher gel strength. This was similar to the results in Zhao et al. [17].

### 2.2. Frequency Sweep Rheological Property

SPI-CG cold-set gels with entanglement networks can be studied with frequency sweep tests, allowing a better understanding of their internal structure and viscoelastic behavior [20]. By monitoring the changes in steady flow behavior, storage modulus (G′), and loss modulus (G″) over time, we evaluated the rheological behavior of SPI cold-set gels with CG added at various concentrations (0–0.30%). 

The G′ and G″ of SPI-CG cold-set gels are shown in Figure 1C,D. From a single sample, SPI-CG cold-set gels showed a greater G′ value than G″ value, indicating a gel-like structure. With the frequency increased, the G′ and G″ of SPI-CG cold-set gels showed a trend of increasing, indicating that they had a certain frequency dependence. With the CG concentration increased, the G′ and G″ of SPI-CG cold-set gels increased first and then decreased. When the addition of CG concentration was 0.20%, the G′ and G″ of SG20 reached a maximum value. It indicated that gels improved in both elasticity and viscosity when CG was added. Previous studies had shown that G′ measures the gel network’s connectivity, which was correlated with cross-linking strength in aggregated microstructures [21]. Therefore, CG might enhance the cross-linking of SPI molecules and act as fillers within the gel network to increase the number of cross-linking sites and enhance resistance to external forces. But the SPI-CG cold-set gel networks might be discontinuous at higher CG concentration, which reduced the number of active chains, leading to G′ and G″ being decreased.

### 2.3. Microstructure

**Figure 2:**
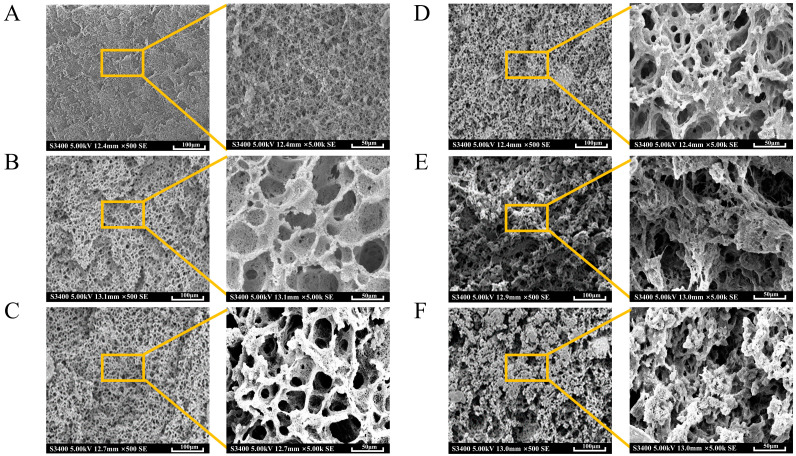
The SEM pictures of SPI-CG cold-set gels. (**A**) SPI. (**B**) SG10. (**C**) SG15. (**D**) SG20. (**E**) SG25. (**F**) SG30.

By SEM, we examined the microstructure of SPI-CG aggregate gels after adding different CG concentrations to illustrate the differences in the gel network structure. Figure 2 shows the microstructure pictures of SPI-CG cold-set gels with different CG concentrations. Figure 2A shows that the SPI structural surface was relatively smooth and connected by filaments. Possibly, this was caused by GDL promoting slow aggregation and cross-linking [22]. Figure 2B–D shows that, after the addition of CG, gel networks transformed into a honeycomb, the entire network skeleton was significantly reinforced, and the gel structure became denser with the increase in CG concentration. This may be due to the fact that CG filled the network voids and promoted intermolecular cross-linking between SPI-SPI by acting as both a filler and cross-linker. A gel network of this type would be stronger and better able to resist external stress, and it would also offer more space for water to be retained through capillary action [23]. When the CG concentration was 0.20%, it contained the densest gel network. It is worth noting that the gels had larger pores than SPI gels after adding CG, which may be related to the electrostatic repulsion between the hydroxyl group of CG and negatively charged SPI molecules. Figure 2E,F shows that when the CG concentration reached 0.25%, the network began to have irregular structures, such as cavities, collapses, etc. This gel network had poor fracture characteristics and poor water-holding capacity [24]. A potential explanation for this could be excess CG resulting in increased molecular distances between SPI chains and interfering with SPI interactions. Meanwhile, when the CG concentration was high, CG molecules became aggregated and unevenly distributed in the gel network, which reduced network continuity and led to a disordered and coarse network structure [25].

### 2.4. Intermolecular Forces

**Figure 3:**
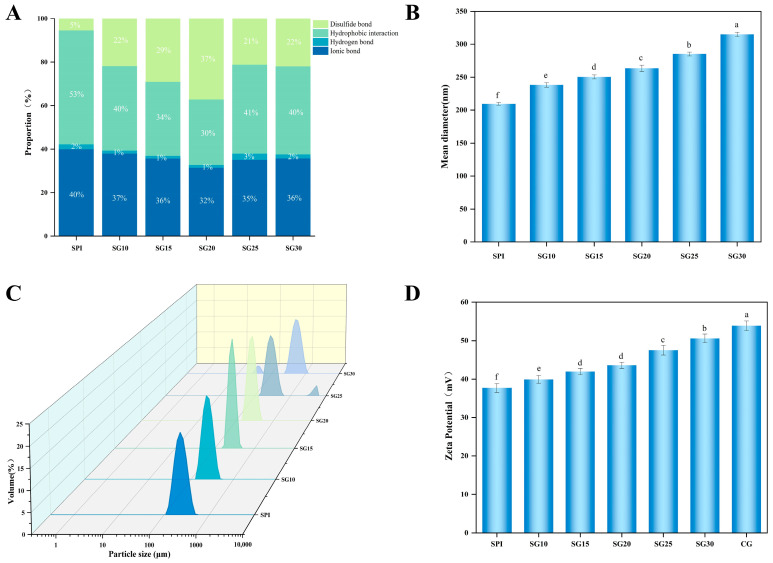
Plotting of (**A**) intermolecular forces of SPI-CG composite gels; (**B**) mean diameter of SPI-CG mixed solutions; (**C**) the particle size distribution of SPI-CG mixed solutions; (**D**) absolute value of Zeta potential of SPI-CG mixed solutions. Data with different letters in (**A**–**D**) are significantly different (*p* < 0.05).

By determining the type and magnitude of the interactions of SPI-CG cold-set gel structures, we can further explore the reason why CG improves the microstructure of SPI cold-set gels. In Figure 3A, the proportions of the intermolecular forces of the SPI-CG cold-set gels are shown. There was a large proportion of all SPI-CG cold-set gels with a hydrophobic interaction, ionic bond, and disulfide bond, which indicated that they were the major forces in SPI-CG cold-set gels. With an increase in CG concentration in SPI-CG cold-set gels, the disulfide bond first increased and then decreased, the hydrophobic interaction first decreased and then increased, and the hydrogen bond and ionic bond showed no difference. As a result, adding CG mainly improved the structure of SPI-CG cold-set gels by changing the proportion of disulfide bond and hydrophobic interactions in SPI-CG cold-set gels. A low concentration of CG may act as a cross-linking agent to promote cross-linking between SH hydrophobic groups of SPI, resulting in forming new disulfide bonds in SPI-CG cold-set gels. This also reduces the hydrophobic groups on the surface of SPI-CG aggregates, therefore weakening the hydrophobic interaction in SPI-CG cold-set gels. However, excessive CG may prevent the cross-linking between SH hydrophobic groups of SPI through self-assembled aggregates, thereby reducing the generation of a disulfide bond and increasing the hydrophobic interaction in SPI-CG cold-set gels. Moreover, when the CG concentration was 0.20%, SG20 had the maximum proportion of disulfide bond and the minimum proportion of hydrophobic interaction. It has been demonstrated that the disulfide bond belonged to a strong bond, which was conducive to forming a network structure with a strong skeleton. This can make the gel structure less easy to be destroyed by external forces, with strong stability [26].

### 2.5. Particle Size of SPI-CG Mixed Solution

The particle size of SPI-CG aggregates formed after the addition of CG was determined in order to assess their size and distribution uniformity. Figure 3B shows that the mean diameter of SPI-CG aggregates increased from 209.00 ± 2.33 nm to 318.30 ± 3.30 nm with an increase in CG concentration (*p* < 0.05). This meant that CG caused large SPI-CG aggregates to form when it was added. This may be due to the fact that the negative charge of the sulfated groups on CG is combined with the positive charge of the amino groups on proteins by electrostatic interactions to form a complex (-OSO_3_^−^-NH_3_^+^) [27]. Figure 3C shows that, when the CG concentration exceeded 0.20%, there was a change from a single peak to a double peak in the particle size distribution. It indicates that SPI-CG aggregates had a non-uniform particle size distribution as a result of the high concentration of CG. This was due to the fact that the SPI binding site was saturated, resulting in aggregates formed between CG-CG. In their study, Wang et al. [28] reported that larger aggregates can be used as precursors for gel formation, which in turn facilitates the formation of the final SPI-CG gel network. However, it is worth noting that too large an SPI-CG aggregate particle size hindered SPI unfolding and cross-linking through the formation of insoluble aggregates, reducing network continuity and adversely affecting the final SPI-CG cold-set gel formed [29]. Moreover, the lack of uniformity in the distribution of particle sizes can impact the organized clustering of the SPI-CG aggregate while forming SPI-CG cold-set gels, consequently influencing their overall structure.

### 2.6. Zeta Potential of SPI-CG Mixed Solution

Zeta potential is a measure of the amount of surface charge on SPI-CG aggregates. They with a larger absolute value of zeta potential represented that they have a higher charge density and stronger electrostatic interactions. Figure 3D shows the zeta potential values of SPI-CG aggregates. All these zeta potential values fell between the pure SPI solution (37.68 ± 1.15 mV) and the pure CG solution (53.86 ± 1.26 mV), confirming the existence of electrostatic interactions in the SPI-CG system [30]. Figure 3D shows that zeta potential absolute values increased with the CG concentration increasing (*p* < 0.05), which indicated that electrostatic interactions among SPI-CG aggregates increased. Everett et al. pointed out that electrostatic interactions strengthen the final protein gel network [31]. Studies showed that a low charge density enhanced the coarseness of gel networks [32]. Due to the lower charge density, there was a greater possibility that SPI aggregates were more unstable than SPI-CG aggregates. When the SPI-CG aggregates were gelled, they settled unevenly, resulting in forming rough gel networks. Thus, adding CG was conducive to forming dense SPI-CG cold-set gel networks by enhancing the charge density.

### 2.7. Gel Strength

**Figure 4:**
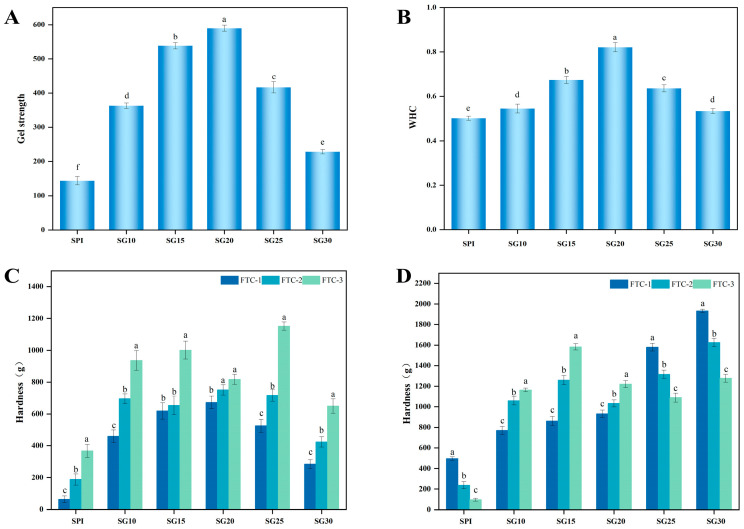
Plotting of (**A**) gel strength; (**B**) WHC; (**C**) freeze–thaw stability; (**D**) steaming stability in SPI-CG composite gels. Data with different letters in (**A**–**D**) are significantly different (*p* < 0.05).

The strength of the gel may affect the texture and quality of the gel, which is essential for food preparation. Figure 4A shows the gel strength of SPI-CG cold-set gels, which showed an overall trend of increasing and then decreasing with the increase in the concentration of CG (*p* < 0.05). The gel strength reached a maximum value of 589.91 ± 8.78 g with the addition of 0.20% CG. Compared with SPI cold-set gel, the gel strength was more than four times greater. A significant increase in the gel strength and mechanical properties of SPI was observed when CG was incorporated. It had been reported that the change in gel strength may be linked to the network structure of SPI-CG cold-set gels and the interaction within the gel matrix between SPI-CG aggregates [16,33]. Thus, the apparent increase in gel strength may be explained by the formation of a firm and ordered network structure and the increased disulfide bond ratio in SPI-CG cold-set gel. When the concentration of CG was high, abnormal aggregates formed and the microstructure of the SPI-CG cold-set gel was disrupted.

### 2.8. Water-Holding Capacity

To function effectively in practical applications, gels must maintain their adsorption and binding qualities in the face of external interference, such as pressures and cuts. By using WHC, it was possible to assess the stability of SPI-CG cold-set gels under a certain pressure [34]. Figure 4B shows that the WHC of the SPI-CG cold-set gels first increased and then decreased with the increase in CG concentration (*p* < 0.05). The WHC reached a maximum value of 89.59 ± 0.02% with the addition of 0.02% CG. It indicates that SPI-CG cold-set gels improved their capacity to retain their original texture after processing. This was consistent with the results of LF-NMR. This may be due to the fact that a gel has a denser and firmer gel network structure after the addition of CG. This promotes the entanglement of water in the network structure and enhances the resistance of SPI-CG cold-set gels to high-speed centrifugation. In addition, this might be due to the abundant hydrophilic groups in CG molecules. It improves some hydrophilic functional groups (OH) on the surface of SPI-CG aggregates, which can bind more water molecules. However, when the CG concentration was high, the gel network became rougher and unable to prevent high-speed centrifugation from damaging the gel, resulting in the water-holding capacity of SPI-CG cold-set gels decreasing.

### 2.9. Freeze–Thaw Stability

Freezing could destroy the original structure of gels, thereby reducing the sensory quality of frozen foods. Therefore, detecting the freezing resistance of SPI-CG cold-set gels was crucial to evaluate their applications in food freezing. We mainly evaluated the ability of SPI-CG cold-set gels to maintain their original performance after freeze–thaw. A small change in the hardness of the SPI-CG cold-set gels represented that their freeze–thaw stability was better. Figure 4C shows the change in the hardness of SPI-CG cold-set gels after three freeze–thaw cycles. The hardness of the samples increased with the number of freeze–thaw cycles. It indicated that freeze–thaw affected the quality of SPI-CG cold-set gels. This was due to the fact that, during the freezing process, the water structure in the SPI-CG cold-set gel system was destroyed, forming ice crystals and making the gel network rough [35]. After thawing, the ice crystals melted away from the gel network, resulting in the water content decreasing in the gel and the content of SPI-CG aggregates increasing [36]. Therefore, the hardness showed an increased trend. It is worth noting that the hardness of SG20 had the minimum change. It indicated that its freeze–thaw stability was the best of all the samples. This may be due to the fact that SG20 had the densest gel network and the lowest water content. It is possible that the kind of gel network could inhibit water movement and migration in an SPI-CG cold-set gel network, resulting in preventing the formation of the large ice crystals that destroy the SPI-CG gel network. Thus, it proved that the addition of CG can enhance the freeze–thaw resistance of SPI cold qualitative gel. It proved that SPI-CG cold-set gel was beneficial to the application of lactone tofu and other related cold chain transportation food.

### 2.10. Steaming Stability

Cooking is an indispensable step in the food processing process and steaming is one of the daily food processing methods. The practical applacation of SPI-CG cold-set gels after freeze–thaw can be further evaluated by their steaming stability. Figure 4D shows the hardness of SPI-CG cold-set gels after three freeze–thaw cycles and steaming treatment. It indicates that, when the number of freeze–thaw cycles increases, the hardness of SG10, SG15, and SG20 show an increasing trend. This may be due to the accelerated massive loss of water in SPI-CG cold-set gels caused by freeze–thaw and high temperatures. When the number of freeze–thaw cycles increases, the hardness of SPI, SG25, and SG30 show a decreasing trend. This may be due to the fact that too much water precipitation could lead to the instability of the gel network structure, resulting in a decrease in gel hardness [37]. After steaming, the hardness of SG20 had the smallest change. This may be due to the fact that, when the CG concentration was 0.20%, during the high-temperature and freezing processes, the gel system lost the least water because of its low water content. Meanwhile, its gel network was the strongest, which can reduce the damage to the gel caused by water precipitation during steaming. Therefore, SPI cold-set gels can be made more stable by adding CG to improve their freeze–thaw and steaming stability.

## 3. Conclusions

In the current study, we focused on the influence of CG concentrations on the textural properties and freeze–thaw stability of SPI cold-set gels. LF-NMR and MRI analysis confirmed that this improvement was due to the fact that the addition of CG improved the ability of gels to constrain water and reduced the water content in the gel system. Rheology confirmed that the SPI-CG cold-set gel with CG added could increase the number of cross-linking sites in the gel network. The microstructure confirmed that SPI-CG cold-set gels formed a uniform and denser three-dimensional network structure as a result of CG altering SPI aggregation behavior. In these experiments, CG added to SPI promoted the formation of SPI-CG cold-set gels’ dense microstructures. Intermolecular interactions analysis confirmed that the addition of CG could form a higher proportion of disulfide bonds by promoting the cross-linking between SPI-SPI molecules. Particle size confirmed that CG promoted the formation of large aggregates. But when the CG concentration exceeded 0.20%, the SPI-CG aggregates were not evenly distributed. Zeta potential confirmed that CG was conducive to enhancing the charge density of SPI-CG mixed solutions. These were the reasons why the microstructures of SPI-CG cold-set gels improved. The characteristics of SPI-CG cold-set gels, including gel strength, water-holding capacity, freeze–thaw stability, and steaming stability, were all significantly affected by the addition of CG. The final results confirmed that the gel with 0.20% CG had the best gel properties. In summary, by adjusting the CG concentration, this research suggests an available strategy for improving the properties and extending the further application of SPI cold-set gels, such as delaying digestion, embedding heat-sensitive nutrients, and for freeze-resistant lactone tofu. With this method, it is possible to meet the freezing needs of SPI cold-set gel transportation and anticipate a wide range of applications in the food industry in the future.

## 4. Materials and Methods

### 4.1. Materials

Commercial soy protein isolate (containing 92.8% protein) was purchased from Shandong Yuwang Ltd. (Shandong, China). Carrageenan (BR grade) was purchased from Source Leaf Biotechnology Co., Ltd. (Beijing, China). The glucose-δ-lactone (GDL) (BR grade) was purchased from Tianjin Fuchen Technology Ltd. (Tianjin, China). All other reagents were analytical grade. In addition, our experiments were conducted with distilled water.

### 4.2. SPI-CG Composite Gels Preparation

The SPI-CG mixed solutions were prepared with 8% SPI and adding different concentrations of CG—0%, 0.10%, 0.15%, 0.20%, 0.25%, 0.30%. After stirring for 2 h, they were hydrated overnight at 4 °C to fully hydrate the proteins. In the following step, all SPI-CG mixed solutions were heated for 30 min in a water bath of 75 °C. After being cooled in an ice-water bath, the samples immediately incorporated 2% GDL and dissolved it. To complete gelation, the samples were left at room temperature for two hours. The samples were named sequentially as SPI, SG10, SG15, SG20, SG25, and SG30.

### 4.3. SPI-CG Composite Gels Preparation Low-Field Nuclear Magnetic Resonance (LF-NMR)

In accordance with Han et al. [38], a nuclear magnetic resonance image analyzer was used to measure the water content of the SPI-CG cold-set gels (MesoMR23-060H-I NMR, SUZHOU NIUMAG Analytical Instrument Co., Ltd., Suzhou, China). We used the CPMG sequence with the following parameters: 21 MHz proton resonance, 3000 echoes (C0), 16 repeat scans (NS), and 0.15 ms half-echo time (TE).

### 4.4. Magnetic Resonance Imaging (MRI)

This study used MRI tests according to Zhang et al. [19] and made a slight modification. MultiExp Inv analysis software (Niumag Electric Co., Shanghai, China) was used to analyze the data. In the MRI measurements, we used a SPIN ECHO sequence to create proton density-weighted images. We chose an intermediate slice with a width of 3 mm (TR (time repetition) = 1500 ms, T sum = 20 ms, SF = 21.255 MHz, mean = 8).

### 4.5. Rheological Properties

The method was modified according to Wang et al. [39]. The rheological properties of the SPI-CG cold-set gels were evaluated using a rotational shear rheometer (TA AR550, TA Instruments, Newcastle, DE, USA) equipped with a 35 mm parallel plate configuration (gap = 1 mm). Prior to testing, the gels were allowed to equilibrate at room temperature for 1 h. The storage modulus (G′) and loss modulus (G″) were determined by performing a frequency sweep at a temperature of 25 °C and a strain of 0.1% within the frequency range of 0.1–15 Hz.

### 4.6. Scanning Electron Microscopy (SEM)

Minor modifications were made to the method described by Wang et al. [40]. The SPI-CG cold-set gel samples were prepared by sectioning them into dimensions of 5 × 5 × 1.5 mm^3^ and then fixing them with glutaraldehyde for a duration of 12 h, followed by rinsing with phosphate buffer. Subsequently, the samples underwent sequential dehydration using alcohol solutions of increasing concentrations (50%, 70%, 90%, and 100% *v*/*v*). Pure tertiary butyl alcohol was then introduced to displace the alcohol within the samples, which were subsequently freeze-dried. Subsequently, ion-sputtering gold plating was performed on the samples. The microstructure of the gel samples was analyzed using a scanning electron microscope (SEM) model S-3400n manufactured by Hitachi Ltd. in Tokyo, Japan. The examination was carried out at magnifications of ×500 and ×5000, with an accelerating voltage of 20 KV.

### 4.7. Intermolecular Forces

In accordance with Lv et al. [41], the intermolecular forces were measured using a modified method. In detail, four dissolving buffers were mixed with 0.5 g of SPI-CG cold-set gels. The solutions contained 0.6 mol/L NaCl, 0.6 mol/L NaCl, and 1.5 mol/L urea, 0.6 mol/L NaCl, and 8 mol/L urea, and 1 mol/L NaOH. They were called A1, A2, A3, and A4, respectively. They were centrifuged at 10,000× *g* for 10 min after homogenizing at 10,000 rpm. As a result, the levels of soluble protein in the supernatants (B1, B2, B3, and B4, respectively) were determined using the method of Bicinchoninic Acid Assay (BCA). The intermolecular forces were calculated as follows:

Ionic bond (%) = B1/B4 × 100(1)

Hydrogen bond (%) = (B2 − B2)/B4 × 100(2)

Hydrophobic interaction (%) = (B3 − B2)/B4 × 100(3)

Covalent bonds (%) = (B4 − B3)/B4 × 100(4)

### 4.8. Particle Size and Zeta Potential

The particle size distributions and zeta potentials were measured, with some modifications, following Huang et al. [42]. By using the Zetasizer Nano ZS 90 instrument (Malvern Instruments Ltd., shanghai, China), mixed SPI-CG solutions with different concentrations of CG were measured. In order to conduct a further analysis, the samples were thinned using distilled water to a concentration of 0.1 mg/mL. A temperature of 25 °C was used for all measurements.

### 4.9. Gel Strength

In accordance with Zhao et al. [43] a texture analyzer (TA. XT Plus C, Stable Micro System Company, Godalming, UK) with a probe of P/0.5 (cylindrical) was used to assess gel strength. Before conducting the assessment, the SPI-CG cold-set gels were permitted to stabilize at room temperature for a duration of 1 h. There were four measurement parameters included: 1.0 mm/s during the pre-test, 2.0 mm/s during the test, and 2.0 mm/s during the post-test. The target depth was 5 mm at the end of the test.

### 4.10. SPI-CG Composite Gels Preparation Water-Holding Capacity (WHC)

The SPI-CG mixed solutions were prepared with 8% SPI and different concentrations. WHC was assessed utilizing the methodology outlined by KOCHER et al. [44]. A 10 mL centrifuge tube was filled with about 5 g of the prepared gel and centrifuged at 8000× *g* for 30 min. Having removed the supernatant, the centrifuged gel was weighed and the WHC calculated. WHC is the weight of the centrifuged gel expressed as a percentage of its original weight. The WHC was calculated as follows:

WHC (%) = (m_2_ − m_0_)/(m_1_ − m_0_) × 100% (5)

Variables m_0_, m_1_, and m_2_ represent the mass of the empty centrifugal tube, the mass of the gel and centrifugation tube combined prior to centrifugation, and the mass of the gel and centrifugation tube combined after centrifugation, respectively.

### 4.11. Texture Profile Analysis (TPA)

A modified version of the method was used to measure the samples prepared in Section 4.12 and Section 4.13, which was developed in accordance with Jin et al. [45]. TPA was performed using a texture analyzer (TA. XT Plus C, Stable Micro System Company, Godalming, UK) with a cylindrical probe of P/0.5. The pre-test, test, and post-test speeds were all 2 mm/s, with a trigger force of 5.0 g.

### 4.12. Freeze–Thaw Stability

The method was modified according to Lai et al. [46]. A freezer was used to freeze all SPI-CG cold-set gels prepared in Section 2.2 for 24 h, and then a thawing chamber was used to thaw them for 4 h at 25 °C. The freeze–thaw cycle (FTC) was repeated three times, and the resulting samples were labeled FTC-1, FTC-2, and FTC-3. The hardness of the samples was determined in Section 4.11 to assess their freeze–thaw stability.

### 4.13. Steaming Stability

All the SPI-CG cold-set gels that were indicated in Section 4.12 were cooked using a steamer basket (MAXCOOK, Guangzhou, China) for 5 min. The hardness of the samples was determined in Section 4.11 to assess their steaming stability.

### 4.14. Statistical Analysis

Each experiment was performed in triplicate, and the results were reported as the mean and standard deviation (SD). The collected data underwent statistical analysis using one-way ANOVA, followed by Duncan’s test, employing SPSS 25 software. Statistics were conducted at a significance level of *p* < 0.05.

## Figures and Tables

**Table 1:** Water content, transverse relaxation times, and corresponding proportions of SPI-CG composite gels.

Simple Name	T_2_	Time of Relaxation (ms)				Water Proportions			
		T_2b_ (ms)	T_2b−1_ (ms)	T_21_ (ms)	T_22_ (ms)	P_2b_ (%)	P_2b−1_ (%)	P_21_ (%)	P_22_ (%)
SPI	16,899.61 ± 77.86 ^a^	0.52 ± 0.09 ^cd^	N.D	109.70 ± 5.01 ^a^	1762.91 ± 28.94 ^a^	2.18 ± 0.10 ^b^	N.D	97.10 ± 0.60 ^a^	0.72 ± 0.08 ^d^
SG10	13,841.79 ± 71.68 ^b^	0.74 ± 0.09 ^bc^	N.D	89.07 ± 2.93 ^b^	1773.75 ± 10.01 ^a^	1.85 ± 0.10 ^c^	N.D	97.06 ± 0.10 ^ab^	0.45 ± 0.11 ^d^
SG15	12,903.93 ± 60.83 ^c^	0.69 ± 0.12 ^bcd^	N.D	83.10 ± 5.17 ^bc^	1162.32 ± 12.12 ^b^	2.49 ± 0.10 ^a^	N.D	96.69 ± 0.26 ^b^	1.33 ± 0.21 ^c^
SG20	11,768.12 ± 126.89 ^e^	0.79 ± 0.14 ^b^	1.70 ± 0.11 ^b^	77.53 ± 4.35 ^c^	943.79 ± 49.42 ^d^	1.98 ± 0.12 ^c^	0.90 ± 0.06 ^b^	94.00 ± 0.20 ^d^	3.84 ± 0.16 ^a^
SG25	12,856.64 ± 118.82 ^c^	1.12 ± 0.10 ^a^	2.77 ± 0.06 ^a^	89.07 ± 6.45 ^b^	1084.37 ± 54.99 ^c^	1.27 ± 0.11 ^d^	1.67 ± 0.75 ^a^	95.26 ± 0.14 ^c^	2.47 ± 0.22 ^b^
SG30	12,371.15 ± 106.05 ^d^	0.52 ± 0.07 ^d^	N.D	83.10 ± 6.90 ^bc^	1080.48 ± 43.66 ^c^	0.61 ± 0.06 ^e^	N.D	95.44 ± 0.15 ^c^	2.79 ± 0.15 ^b^

Notes: a–e, different lowercase letters in the same column indicate statistically significant differences at *p* < 0.05; N.D, not detectable; T_2,_ the relaxation time peak areas; T_2b_, T_2b−1_, T_21_, and T_22_, segmented relaxation times which represented bound water, immobile water, immobile water, and free water, respectively; P_2b_, P_2b−1_, P_21_, and P_22_, the proportions of segmented relaxation times which represented bound water, immobile water, immobile water, and free water, respectively.

## Data Availability

The raw data supporting the conclusions of this article will be made available by the authors on request.
